# Dynamics of the ACE2–SARS-CoV-2/SARS-CoV spike protein interface reveal unique mechanisms

**DOI:** 10.1038/s41598-020-71188-3

**Published:** 2020-08-26

**Authors:** Amanat Ali, Ranjit Vijayan

**Affiliations:** Department of Biology, College of Science, United Arab Emirates University, PO Box 15551, Al Ain, United Arab Emirates

**Keywords:** Viral proteins, SARS-CoV-2, SARS virus, Molecular modelling

## Abstract

The coronavirus disease 2019 (COVID-19) pandemic, caused by the severe acute respiratory syndrome coronavirus 2 (SARS-CoV-2), is a major public health concern. A handful of static structures now provide molecular insights into how SARS-CoV-2 and SARS-CoV interact with its host target, which is the angiotensin converting enzyme 2 (ACE2). Molecular recognition, binding and function are dynamic processes. To evaluate this, multiple 500 ns or 1 μs all-atom molecular dynamics simulations were performed to better understand the structural stability and interfacial interactions between the receptor binding domain of the spike (S) protein of SARS-CoV-2 and SARS-CoV bound to ACE2. Several contacts were observed to form, break and reform in the interface during the simulations. Our results indicate that SARS-CoV-2 and SARS-CoV utilizes unique strategies to achieve stable binding to ACE2. Several differences were observed between the residues of SARS-CoV-2 and SARS-CoV that consistently interacted with ACE2. Notably, a stable salt bridge between Lys417 of SARS-CoV-2 S protein and Asp30 of ACE2 as well as three stable hydrogen bonds between Tyr449, Gln493 and Gln498 of SARS-CoV-2 and Asp38, Glu35 and Lys353 of ACE2 were observed, which were absent in the ACE2–SARS-CoV interface. Some previously reported residues, which were suggested to enhance the binding affinity of SARS-CoV-2, were not observed to form stable interactions in these simulations. Molecular mechanics-generalized Born surface area based free energy of binding was observed to be higher for SARS-CoV-2 in all simulations. Stable binding to the host receptor is crucial for virus entry. Therefore, special consideration should be given to these stable interactions while designing potential drugs and treatment modalities to target or disrupt this interface.

## Introduction

The recent outbreak of coronavirus disease 2019 (COVID-19), caused by the novel severe acute respiratory syndrome coronavirus 2 (SARS-CoV-2), has affected all walks of life. Genomic studies have established that SARS-CoV-2 belong to the betacoronavirus genus, which also includes SARS-CoV and MERS-CoV that were associated with previous outbreaks of relatively smaller scale^1–3^. These coronaviruses attach to the host cell with the aid of the spike (S) glycoprotein present on its envelope. Coronavirus S glycoprotein is composed of two subunits—the S1 subunit is important for binding to the host cell receptor and the S2 subunit is responsible for the fusion of the virus and the host cell’s membrane. Angiotensin converting enzyme 2 (ACE2), an enzyme located on the outer surface of a wide variety of cells, is the primary host cell target with which the S protein of SARS-CoV and SARS-CoV-2 associates^4–6^. The receptor binding domain (RBD) of the S1 subunit of these viruses binds to the outer surface of the claw like structure of ACE2^7^.

**Figure 1:**
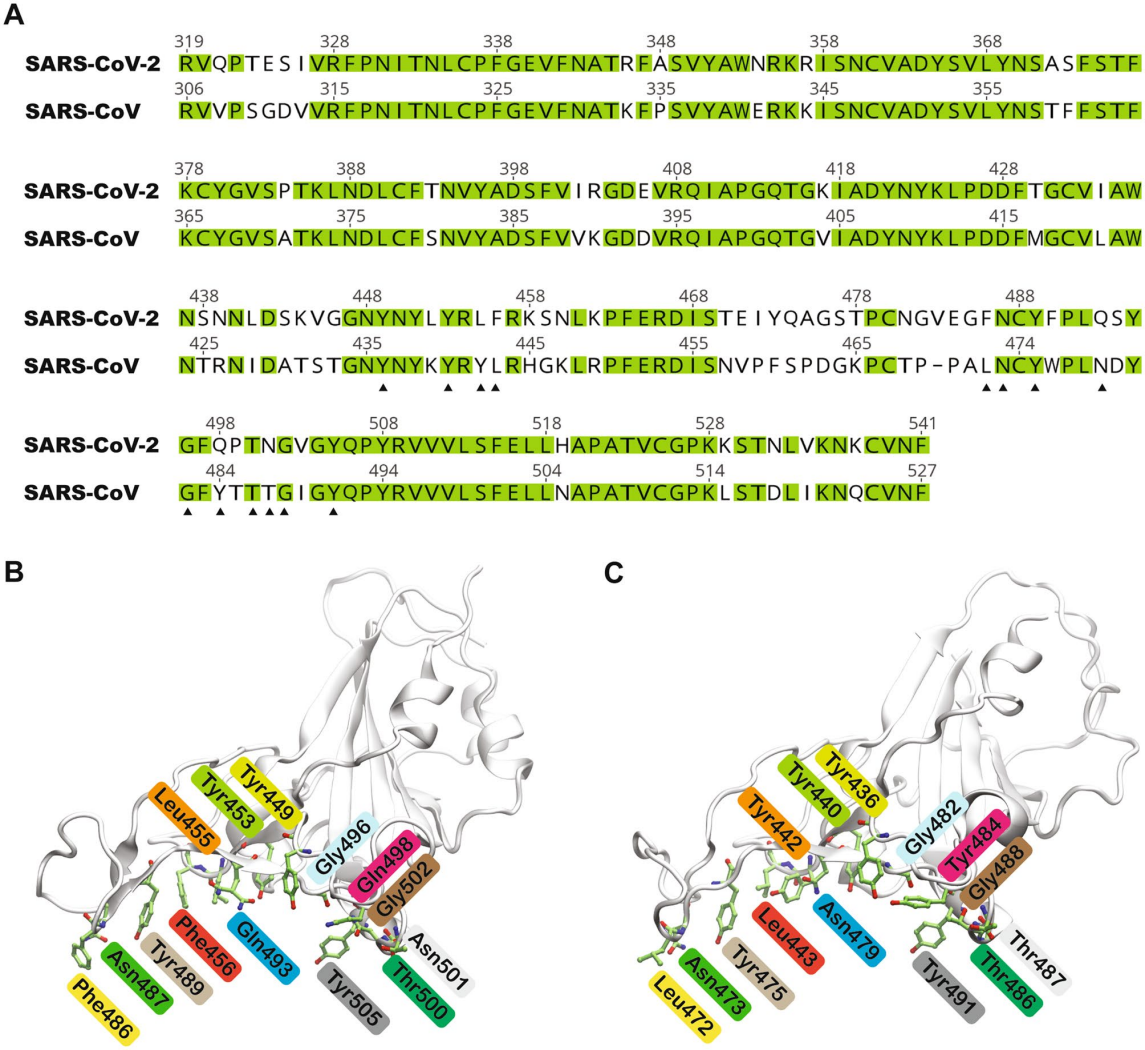
Comparison of the S protein RBD of SARS-CoV-2 and SARS-CoV. (**A**) Sequence alignment of the SARS-CoV-2 and SARS-CoV S protein RBD. Sequences were obtained from UniProt (https://www.uniprot.org) with accession numbers P0DTC2 (SARS-CoV-2 S glycoprotein) and P59594 (SARS-CoV S glycoprotein), and aligned using Geneious 9.1.2 (https://www.geneious.com). Conserved residues are highlighted in green. Residues reported to interact with ACE2 are marked with a filled triangle. (**B**) Structure of the SARS-CoV-2 S protein RBD (PDB ID: 6M0J, chain E) with the fourteen residues shown in green stick representation. (**C**) Structure of the SARS-CoV S protein RBD (PDB ID: 2AJF, chain E) with the fourteen residues shown in green stick representation. Residues in equivalent positions in SARS-CoV-2 and SARS-CoV are labelled with the same colour.

The sequence similarity of the RBD region of SARS-CoV and SARS-CoV-2 S protein is between 73 and 76%^7^. Fourteen residues of the SARS-CoV S protein RBD have been reported to interact with human ACE2 (Fig. 1). These are Tyr436, Tyr440, Tyr442, Leu443, Leu472, Asn473, Tyr475, Asn479, Gly482, Tyr484, Thr486, Thr487, Gly488 and Tyr491^8,9^. Only eight of these residues are conserved in SARS-CoV-2^9^. The equivalent conserved residues in SARS-CoV-2 are Tyr449, Tyr453, Asn487, Tyr489, Gly496, Thr500, Gly502, and Tyr505, while Leu455, Phe456, Phe486, Gln493, Gln498 and Asn501 are substituted. Recent studies have highlighted that SARS-CoV-2 S RBD binds to ACE2 with a higher binding affinity compared to SARS-CoV S RBD^10,11^. This was linked to five substituted residues. Importantly, SARS-CoV-2 residues Gln493 and Asn501 (Asn479 and Thr487 in SARS-CoV) are located near viral binding hotspot residues Lys31 and Lys353 on human ACE2. It has been reported that SARS-CoV-2 residues Gln493 and Asn501 target these hotspot residues more efficiently than SARS-CoV^7^. Additionally, Leu455, Phe486 and Gln498 of SARS-CoV-2 have also been suggested to interact with these hotspot residues more favourably than equivalent residues of SARS-CoV, thereby enhancing the ability of SARS-CoV-2 to bind to ACE2^7^. Hence, the mutated residues interact more favourably with the viral hotspot residues and contribute more to the binding of SARS-CoV-2 to ACE2 when compared to SARS-CoV^7,11^. However, the stability of these interactions have not been clearly elucidated.

Evaluation of the binding of SARS-CoV-2 and SARS-CoV RBDs with ACE2 have revealed more hydrogen bonds and electrostatic interactions in SARS-CoV-2^10,12^. These findings are consistent with significantly lower equilibrium dissociation constant (*K*_D_) obtained in in vitro binding assays of SARS-CoV-2 when compared to SARS-CoV^9,13^. However, very little is known about the stability and contribution of polar and hydrophobic contacts formed between SARS-CoV-2/SARS-CoV and ACE2. To address this, several long molecular dynamics (MD) simulations and in silico binding free energy calculations were performed using multiple structures of SARS-CoV-2/SARS-CoV2 S protein RBD bound to ACE2.

Atomic level three-dimensional structure of several SARS-CoV-2 proteins have now been determined^14–17^. These X-ray crystallography based structures provide remarkable insights into macromolecular structure and intermolecular interactions. However, molecular recognition and binding are dynamic processes. MD simulations often complement traditional structural studies for looking at the dynamics of these processes at the atomic level^18,19^. Such simulations can provide insights into the structural stability of macromolecular complexes, flexibility of interacting subunits and the interactions of residues in the interface.

Here, we report the stability, binding dynamics and energetics of SARS-CoV-2 S RBD bound to ACE2, and compare this with the dynamics of SARS-CoV S RBD, by running multiple 500 ns and 1 μs all-atom MD simulations. High resolution X-ray crystal structures of SARS-CoV-2/SARS-CoV S RBD bound to ACE2 illustrate fourteen positions (Fig. 1) that are associated with the interaction between SARS-CoV/SARS-CoV-2 and ACE2. The primary objective of this study was to identify both similarities and dissimilarities in the dynamics of the interactions between SARS-CoV-2/SARS-CoV and ACE2 and to identify key residues that could be vital to the integrity of this interface. This would provide insights into residues that could be targeted for disrupting this interface.

## Results

### MD simulations of ACE2–SARS-CoV-2 and ACE2–SARS-CoV complexes

Five hundred nanosecond MD simulations of two ACE2–SARS-CoV-2 structures (PDB IDs: 6M0J and 6LZG) were performed in triplicate to ensure that results were not biased by a single structure. One of the 6M0J simulations was extended to 1 μs to ensure that the interactions were faithfully retained for a longer duration. In the case of ACE2–SARS-CoV complex, two sets of 500 ns simulations were performed using chains A (ACE2) with E (S) and B (ACE2) with F (S) from the structure 2AJF. Three 500 ns simulations were performed using A/E chains and two with B/F. To observe the differences seen in the simulations for a longer period, one simulation each of the A/E and B/F complexes were further extended to 1 μs.

**Figure 2:**
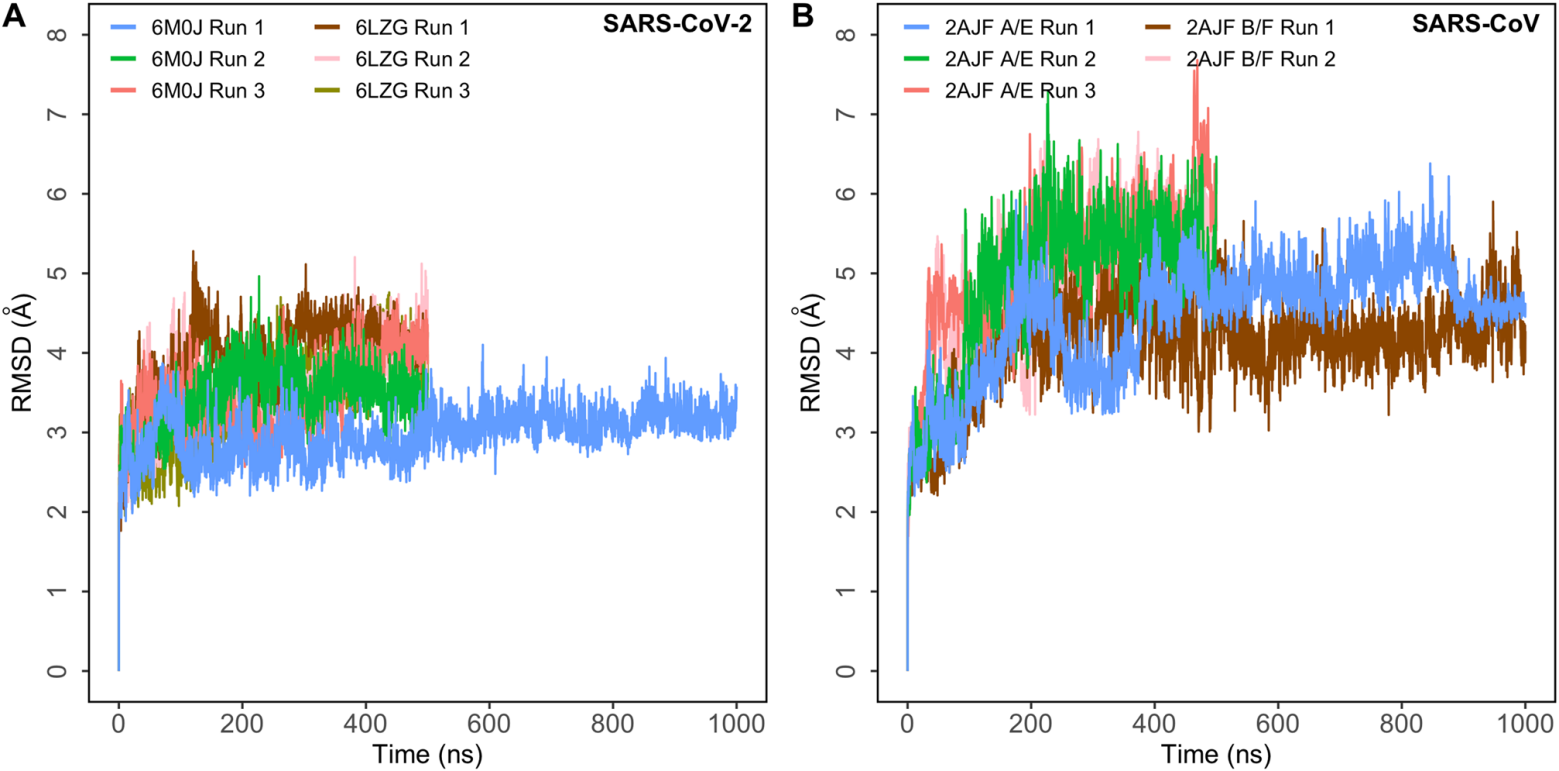
Root mean square deviation (RMSD) of protein Cα atoms with respect to the initial structure obtained from six independent runs (6M0J: 1 × 1 μs and 2 × 500 ns; 6LZG: 3 × 500 ns) of SARS-CoV-2 S and five independent runs (2AJF A/E: 1 × 1 μs and 2 × 500 ns; 2AJF B/F: 1 × 1 μs and 1 × 500 ns) of SARS-CoV S bound to ACE2. (**A**) Simulations of ACE2–SARS-CoV-2 complex; (**B**) simulations of ACE2–SARS-CoV complex.

The overall structural integrity of all simulations of ACE2–SARS-CoV2 complex was retained with a Cα root mean square deviation (RMSD) from the starting structure that was less than 4.5 Å (Fig. 2A). In the case of the ACE2–SARS-CoV simulations, the structures had a Cα RMSD that was under 6 Å (Fig. 2B). The oscillating RMSD of the first ACE2–SARS-CoV simulation was characteristic of the closing/opening motion of the ACE2 claw like structure^20^. A higher RMSD in the SARS-CoV simulations was associated with the detachment of the S protein from one end of the interface in four simulations. However, the other end remained attached as evident from specific contacts that were retained throughout the simulations. To study this further, two simulations, one in which the S protein remained stably bound and the other in which one end detached, were extended from 500 ns to 1 μs.

Protein secondary structure composition and compactness, as indicated by the radius of gyration, of ACE2 and S protein structures were also preserved throughout the simulations (Supplementary Figure S1).

### Comparison of regional fluctuations in the ACE2–SARS-CoV-2 and ACE2–SARS-CoV complexes

**Figure 3:**
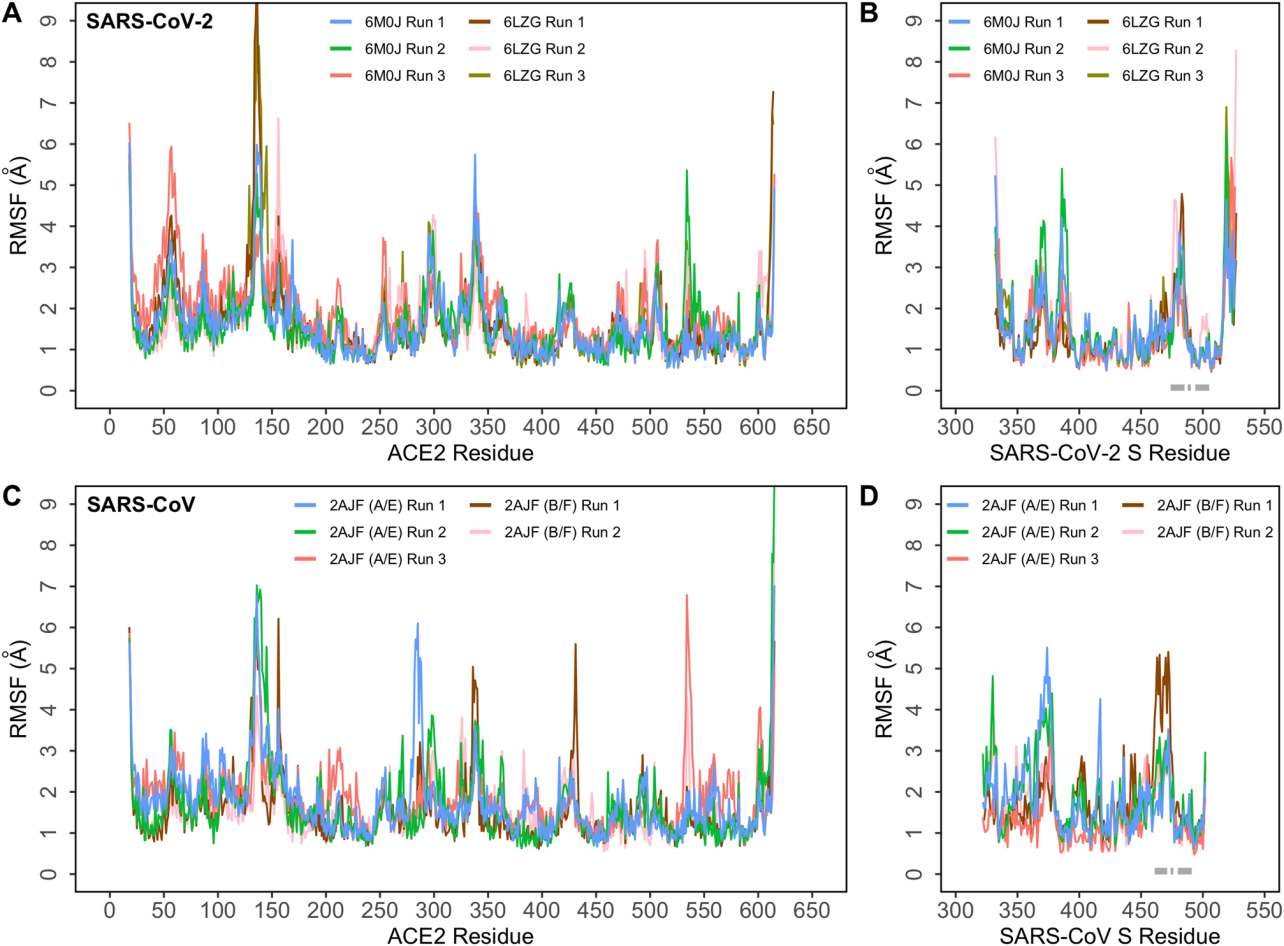
Root mean square fluctuation (RMSF) of protein Cα atoms obtained from six independent runs (6M0J: 1 × 1 μs and 2 × 500 ns; 6LZG: 3 × 500 ns) of SARS-CoV-2 S and five independent runs (2AJF A/E: 1 × 1 μs and 2 × 500 ns; 2AJF B/F: 1 × 1 μs and 1 × 500 ns) of SARS-CoV S bound to ACE2. (**A**) RMSF of Cα atoms of ACE2 protein in the ACE2–SARS-CoV-2 complex; (**B**) RMSF of Cα atoms of SARS-CoV-2 S RBD in the ACE2–SARS-CoV-2 complex; (**C**) RMSF of Cα atoms of ACE2 protein in the ACE2–SARS-CoV complex; (**D**) RMSF of Cα atoms of SARS-CoV S RBD in the ACE2–SARS-CoV complex. Loop regions of SARS-CoV-2 and SARS-CoV S RBD that make contact with ACE2 are identified with grey bars.

To identify and compare backbone stability and fluctuations of the two complexes, root mean square fluctuation (RMSF) of backbone Cα atoms were computed and plotted (Fig. 3). This was also projected as beta factors in PDB structures and visualized (Supplementary Figure S2). Three loops (residues 474–485, 488–490, and 494–505) of the SARS-CoV-2 S protein RBD make contact with ACE2. The homologous region in SARS-CoV range between 461–471, 474–476, and 480–491. In the ACE2–SARS-CoV-2 simulations, loops formed by residues 474–485 and 488–490 exhibited a comparatively higher fluctuation with respect to the rest of the RBD structure while the 494–505 loop demonstrated very limited fluctuations (Fig. 3B) However, in ACE2–SARS-CoV simulations, both loops demonstrated higher fluctuations throughout the simulations (Fig. 3D). Interestingly, the region around Lys417 showed very limited fluctuations in SARS-CoV-2. Overall, the backbone of SARS-CoV-2 S RBD exhibited lower fluctuations compared to SARS-CoV (Fig. 3B,D).

S protein RBD structures were extracted from the SARS-CoV-2 and SARS-CoV complexes and simulated independently to study how the RBD structures behave when not bound to ACE2. The RMSF of the RBD-only structures were then compared with the corresponding RBD bound to ACE2. Interestingly, in SARS-CoV-2 S RBD, residues between 469 and 505, that form loops, exhibited higher fluctuations when not bound to ACE2 (Supplementary Figure S3A). The equivalent region of the SARS-CoV S RBD did not produce such a marked difference in bound and unbound simulations (Supplementary Figure S3B). It would appear that this region in SARS-CoV-2 RBD is more effectively stabilized after binding to ACE2. Several residues between 484 and 505 are vital to the ACE2–S RBD interface. The loop region between residues 384–392 in SARS-CoV-2 fluctuated more when bound to ACE2, while there was no noticeable difference in the SARS-CoV simulations (Supplementary Figures S3A and S3B). This loop is not located in the interface and, thus, it is not clear if this has any significance.

To look at the effect of SARS-CoV-2 and SARS-CoV binding on backbone stability and residue fluctuations of ACE2, the root mean square fluctuation (RMSF) of backbone Cα atoms of ACE2 were evaluated (Fig. 3A,C). The fluctuation of ACE2 backbone in both SARS-CoV-2 and SARS-CoV complexes were comparable and showed limited fluctuations except in a loop region (278–291) where the SARS-CoV-2 bound complex showed lower fluctuations compared to SARS-CoV bound complex (Fig. 3A,C). More importantly, residues in the interfacial region of ACE2 (residues 78–83 and 353–357) exhibited slightly lower fluctuations in SARS-CoV-2. Additionally, loop regions of ACE2—82–89 and 351–354—also exhibited slightly lower fluctuations in SARS-CoV-2 (Fig. 3A,C).

### Interfacial residue contact duration differs substantially between SARS-CoV-2 and SARS-CoV bound complexes

**Figure 4:**
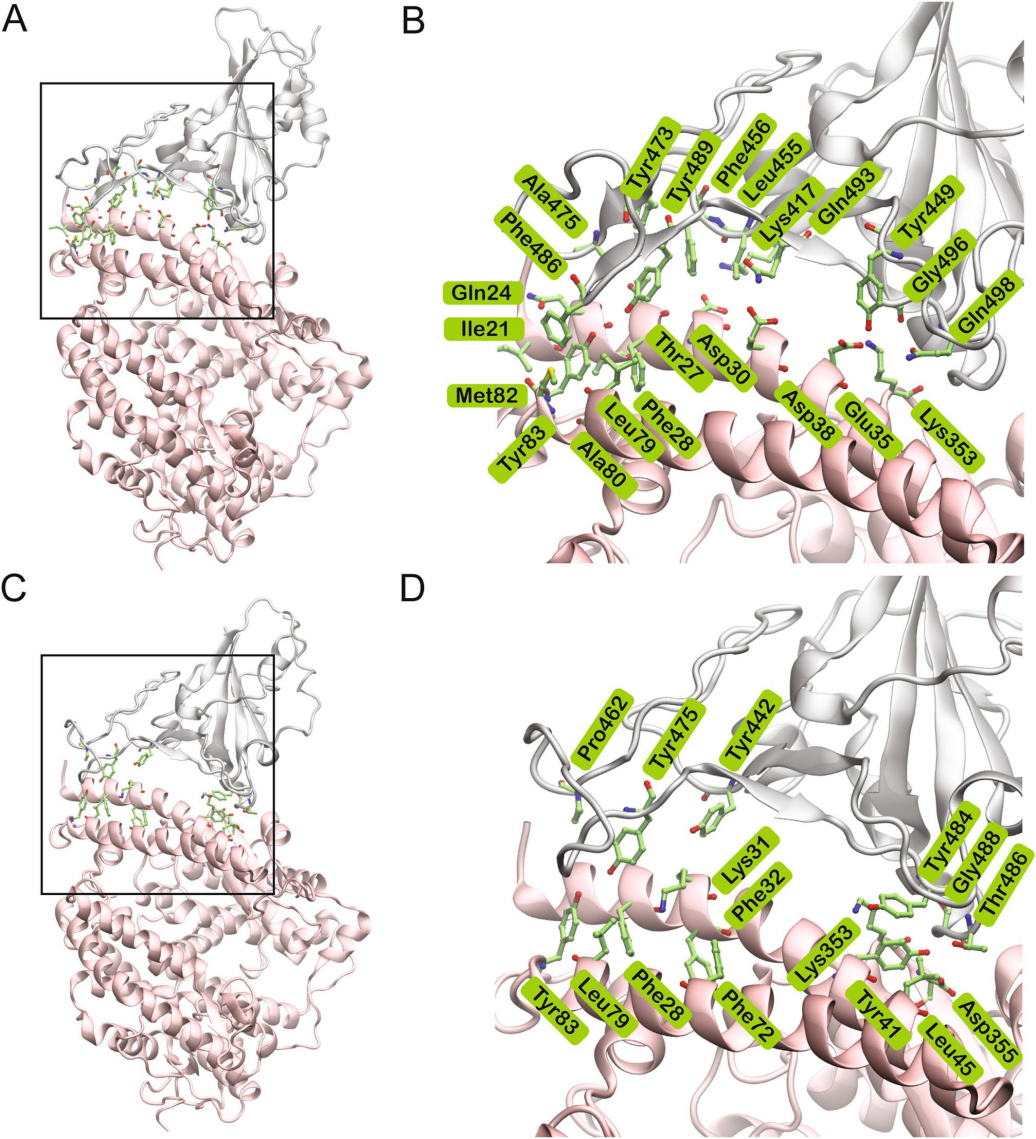
Structures of the SARS-CoV-2 (PDB ID: 6M0J) and SARS-CoV (PDB ID: 2AJF) S RBD (grey) bound to ACE2 (pink). The interface is enlarged in the adjacent image showing the interacting residues in green stick representation. (**A**) SARS-CoV-2 S protein RBD (grey) bound to ACE2 (pink); (**B**) The boxed region in A is enlarged showing the residues that interact in the interface. (**C**) SARS-CoV S protein RBD (grey) bound to ACE2 (pink); (**D**) The boxed region in C is enlarged showing the residues that interact in the interface.

**Figure 5:**
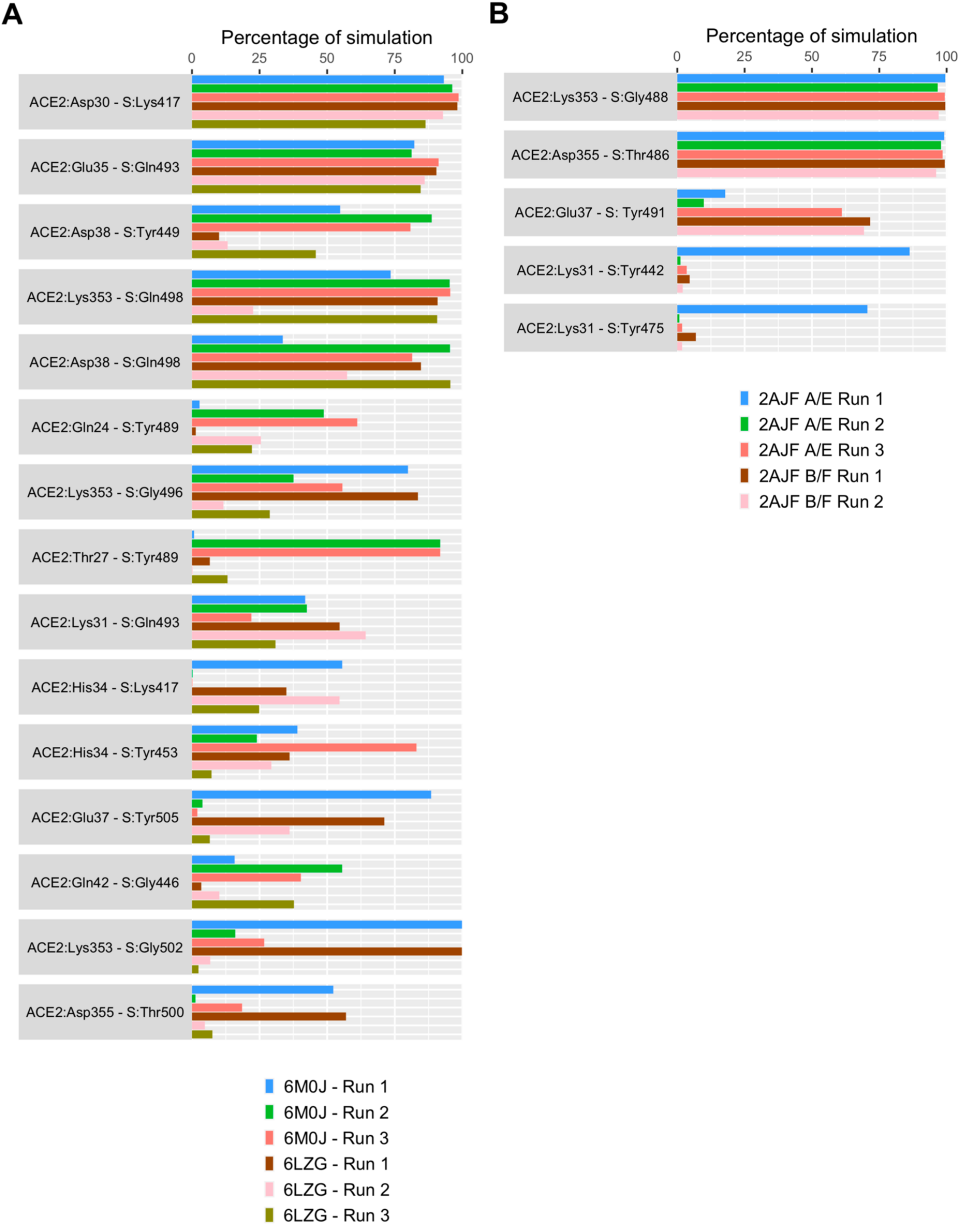
The percentage of simulation time during which intermolecular polar contacts were retained between ACE2 and SARS-CoV-2/SARS-CoV S protein RBD residues. (**A**) Intermolecular contacts from six independent runs of ACE2–SARS-CoV-2 structures (6M0J: 1 × 1 μs and 2 × 500 ns; 6LZG: 3 × 500 ns); (**B**) Intermolecular contacts from five independent runs of ACE2–SARS-CoV structures (2AJF A/E: 1 × 1 μs and 2 × 500 ns; 2AJF B/F: 1 × 1 μs and 1 × 500 ns).

Several intermolecular contacts were observed to form, break and reform during the simulations (Fig. 4). This included hydrophobic interactions, salt bridges, hydrogen bonds, π–π and cation–π interactions (Supplementary Tables S1 and S2). Some of these interactions were more persistent than others. The residues of SARS-CoV-2/SARS-CoV S RBD that formed consistent polar interactions with ACE2 are shown in Fig. 5A,B. The duration of specific intermolecular contacts between SARS-CoV-2/SARS-CoV and ACE2 interfaces and the dynamics of the salient ones along the length of the simulation trajectories are shown in Supplementary Table S1 and Supplementary Figures S4–S7.

Lys31 and Lys353 of ACE2 normally forms intramolecular salt bridges with Glu35 and Asp38 and are buried in a hydrophobic environment^4,8^. In the SARS-CoV simulation where the S RBD remained stably bound to ACE2 (2AJF A/E Run 1), the Lys31 hotspot was observed to consistently form cation–π interactions with Tyr442 and Tyr475 of SARS-CoV S RBD (Fig. 5B). However, Leu455 and Tyr489, the residues at equivalent positions in SARS-CoV-2 did not interact with Lys31 in any of the SARS-CoV-2 simulations (Supplementary Table S1). Instead, Glu484 and Gln493 of SARS-CoV-2 formed intermittent interactions with Lys31 (Supplementary Table S1). The likelihood of an interaction between Gln493 of SARS-CoV-2 and Lys31 residue of ACE2 was reported recently^7^.

Next, the backbone of Gly488 of SARS-CoV consistently interacted with Lys353 of ACE2 for almost the full duration of all five simulations, while Gly502 at the equivalent location in SARS-CoV-2 formed sustained interactions with Lys353 of ACE2 in only one simulation each of structures 6M0J and 6LZG (Fig. 5A,B). Additionally, the side chain of Gln498 of SARS-CoV-2 formed sustained interactions with Lys353 of ACE2 in all simulations, while the backbone of Gly496 of SARS-CoV-2 S RBD of structures 6M0J and 6LZG formed sustained interactions in two and one simulations, respectively, with Lys353 of ACE2. Such an interaction was absent in equivalent residues of SARS-CoV simulations (Fig. 5A,B). This suggests that SARS-CoV-2 and SARS-CoV differ in how they target the basic Lys31 and Lys353 residues of ACE2.

Tyr449, a conserved residue of SARS-CoV-2 S RBD interacted with Asp38 of ACE2 in 6M0J simulations. However, this interaction was observed in only one simulation of 6LZG (Fig. 5A and Supplementary Figures S4C and S5C). Tyr489, another conserved residue of SARS-CoV-2, consistently interacted with Gln24 of ACE2 in two simulations of 6M0J, while this interaction did not appear to be stable in 6LZG simulations (Fig. 5A and Supplementary Figures S4F and S5F). Similar interactions were not observed in SARS-CoV (Supplementary Table S1). Tyr453 and Tyr505 of SARS-CoV-2 were observed to interact with His34 and Gln37, respectively, of ACE2 in only one simulation run of each structure (Fig. 5A). Corresponding residues in SARS-CoV did not appear to form such sustained interactions except Tyr491 which consistently interacted with Gln37 in three out of five simulations (Fig. 5B). Notably, Gln493 and Gln498 of SARS-CoV-2 showed sustained interactions with Glu35 and Asp38, respectively, in all simulations (Fig. 5A and Supplementary Figures S4B, S4E, S5B and S5E) while the corresponding Asn479 and Tyr484 in SARS-CoV exhibited extremely weak interactions (Supplementary Table S1). This is in agreement with recent work that showed the likely existence of Tyr449–Asp38, Tyr453–His34, Tyr489–Gln24, and Gln493–Glu35 interactions between SARS-CoV-2 S RBD and ACE2^11, 20, 21^.

Among the mutated residues in SARS-CoV-2, Lys417 consistently interacted with Asp30 of ACE2 in all simulations of 6M0J and 6LZG (Fig. 5A and Supplementary Figures S4A and S5A). However, Ala475 and Gly446 residues of SARS-CoV-2 S RBD formed marginally better intermittent interactions with Gln24 and Gln42, respectively, in 6M0J when compared to 6LZG (Supplementary Table S1). These interactions were not observed in corresponding residues of SARS-CoV (Supplementary Table S1). Significantly, a very strong salt bridge was established and sustained between Lys417 of SARS-CoV-2 spike RBD and Asp30 of ACE2 for nearly the full duration of all simulations (Fig. 5A and Supplementary Figures S4A and S5A). Notably, this salt bridge is absent in SARS-CoV since the equivalent residue is Val404, which is incapable of forming such an interaction (Supplementary Table S1).

To look at the dynamics of the interface, interactions that were maintained for at least 50% of the total simulation time in at least three simulations of the two complexes were evaluated. Four interfacial residues in SARS-CoV-2 (Lys417, Gln493, Tyr449 and Gln498) and three in SARS-CoV (Thr486, Gly488 and Tyr491) maintained such interactions with four (Asp30, Glu35, Asp38 and Lys353) and three (Glu37, Lys353 and Asp355) residues of ACE2 respectively (Fig. 5A,B). Hence, there are noteworthy differences between how the two viral spike proteins interact with ACE2 and the larger number of sustained interactions in SARS-CoV-2 spike protein could be associated with a higher binding affinity of SARS-CoV-2.

The total number of intermolecular hydrogen bonds between the two complexes were monitored throughout the simulation. A higher number of hydrogen bonds were observed in both structures of the ACE2–SARS-CoV-2 complex (mean ± SD for 6 simulations: 10.9 ± 2.0, 12.2 ± 1.9, 11.6 ± 1.8, 11.4 ± 1.9, 8.6 ± 2.9 and 11.5 ± 2.0) when compared to ACE2–SARS-CoV complex (4.0 ± 1.5, 3.4 ± 2.1, 3.7 ± 1.4, 5.1 ± 1.8 and 5.1 ± 1.6) (Supplementary Figure S8). A consistently higher number of hydrogen bonds formed between ACE2 and SARS-CoV-2 S RBD is in agreement with previous studies^12^. This would also contribute to the binding affinity between SARS-CoV-2 RBD and ACE2 and supports previous in vitro studies that demonstrated a better binding affinity of SARS-CoV-2 for ACE2 when compared to SARS-CoV^10,11,13^.

Contact distance of the most stable interfacial residues of SARS-CoV-2 and SARS-CoV were also evaluated and plotted since this gives a better indication of interactions rather than cutoffs used for determination of an interaction. The contact distance density plot of consistently interacting residues showed sharper peaks (Supplementary Figures S4–S7). Fewer interactions and hydrogen bonds in the SARS-CoV bound complex was due to the notable separation of the RBD from one end of the interface near the Lys31 residue of ACE2 (Figs. 4, 5 and Supplementary Figure S2).

Recent studies, based on models of the SARS-CoV-2 S protein RBD, have indicated that Leu455, Phe486, Ser494 and Asn501 of SARS-CoV-2 are important for binding to ACE2 via their interaction with Met82, Tyr83, Lys31 and Tyr41 residues^3,7,11^. However, in simulations, only a weak π–π interaction was observed between Phe486 and Tyr83 (Supplementary Table S1). While, Leu455, Ser494 and Asn501 were not observed to form any significant interactions with ACE2.

Stability of hydrophobic interactions between SARS-CoV-2/SARS-CoV and ACE2 was also monitored (Supplementary Table S2). Residues that were involved in hydrophobic interactions are included in Fig. 4. SARS-CoV-2 S RBD formed more consistent hydrophobic contacts with ACE2 compared to SARS-CoV S RBD. The existence of higher number of consistent hydrophobic contacts between SARS-CoV-2 and ACE2 also contributes to a higher binding affinity of SARS-CoV-2 for ACE2. The N-terminus of ACE2 formed consistent hydrophobic interactions with SARS-CoV-2 S RBD. Leu79, Met82 and Tyr83 of ACE2 exhibited consistent contact with Phe486 of SARS-CoV-2 S in simulations of both 6M0J and 6LZG. Leu472 of the SARS-CoV, which corresponds to Phe486 of SARS-CoV-2 did not exhibit such interactions. This is noteworthy since previous studies had reported that mutating Leu472 to Phe486 in SARS-CoV-2 contributes to the enhanced binding affinity of SARS-CoV-2 for ACE2^7,10^. Tyr489 of SARS-CoV-2 showed consistent contacts with Phe28 and Tyr83 of ACE2 (Fig. 4) in all simulations. The conserved Tyr475 of SARS-CoV, which is equivalent to Tyr489 of SARS-CoV-2, formed very weak contacts with both Phe28 and Tyr83 of ACE2. However, Tyr475 of SARS-CoV was observed to form sustained contacts with Phe32 and Phe72 of ACE2 in the run where the interface remained intact. Fewer hydrophobic contacts observed in the other simulations is due to the separation of SARS-CoV S RBD from the N-terminus of ACE2. Thr27 of ACE2 interacted with Leu455, Phe456, Tyr473, Ala475 and Tyr489 for the entire duration of all SARS-CoV-2 simulations. Interestingly, all but Tyr489 have been mutated in SARS-CoV-2 and perhaps enables it to bind more strongly. These interactions were not observed in any of the ACE2–SARS-CoV simulations. Tyr484 of SARS-CoV formed sustained hydrophobic contacts with Tyr41 and Leu45 of ACE2 in most of the simulations. A polar Gln498 replaces Tyr484 in SARS-CoV-2. Interestingly, Gln498 of SARS-CoV-2 was observed to form consistent hydrogen bonds with Asp38 and Lys353 in both structures of SARS-CoV-2 (Fig. 5A).

An analysis of both polar and hydrophobic interactions clearly reveal that SARS-CoV-2 S RBD formed a larger number of interactions with ACE2 compared to SARS-CoV RBD (Fig. 5 and Supplementary Tables S1 and S2). This could explain the higher affinity of SARS-CoV-2 than SARS-CoV for ACE2. To look at energetic contributions, free energy of binding (ΔG_bind_) of SARS-CoV-2 and SARS-CoV S RBDs to ACE2 was estimated from frames of all MD simulations using molecular mechanics-generalized Born surface area (MM-GBSA) approach. All six SARS-CoV-2 S RBD bound simulations exhibited ΔG_bind_ values (−118.2 ± 13.5 kcal/mol, −137.9 ± 11 kcal/mol, −140.0 ± 10.6 kcal/mol, −121.5 ± 12.7 kcal/mol, −106.5 ± 24.8 kcal/mol, and −133.4 ± 11.3 kcal/mol) that were substantially higher than SARS-CoV S bound RBD simulations (−71.2 ± 15.4 kcal/mol, −50.2 ± 29.7, −44.5 ± 17.6 kcal/mol, −58.9 ± 18.7 kcal/mol and −56.9 ± 17.9). This is in agreement with the higher binding affinity reported for SARS-CoV-2. Interestingly, the first SARS-CoV simulation, where the entire interface was intact, exhibited a higher ΔG_bind_ than the simulations where part of the interface was disrupted. However, the still substantial ΔG_bind_ supports the ability of the S protein to stay partially bound to ACE2. Hydrophobic contribution to ΔG_bind_ was also higher for SARS-CoV-2 S RBD (−30.8 ± 3.9 kcal/mol, −37.9 ± 3.2 kcal/mol, −38.7 ± 3.0 kcal/mol, −32.6 ± 4.2 kcal/mol, −33.3 ± 6.7 kcal/mol, and −39.6 ± 3.8 kcal/mol) when compared to SARS-CoV S RBD (−23.6 ± 5.9 kcal/mol, −14.5 ± 10.1 kcal/mol, −13.0 ± 6.7 kcal/mol, −17.7 ± 6.0 kcal/mol, and −17.3 ± 5.5 kcal/mol). Hydrophobic contributions are also higher in the case of SARS-CoV-2 than SARS-CoV. In the case of SARS-CoV, a drop in this contribution can be observed when it breaks hydrophobic interactions at the N-terminus of ACE2. Due to the approximations and assumptions employed in MM-GBSA calculations, ΔG_bind_ values obtained here should be interpreted qualitatively rather than quantitatively.

**Figure 6:**
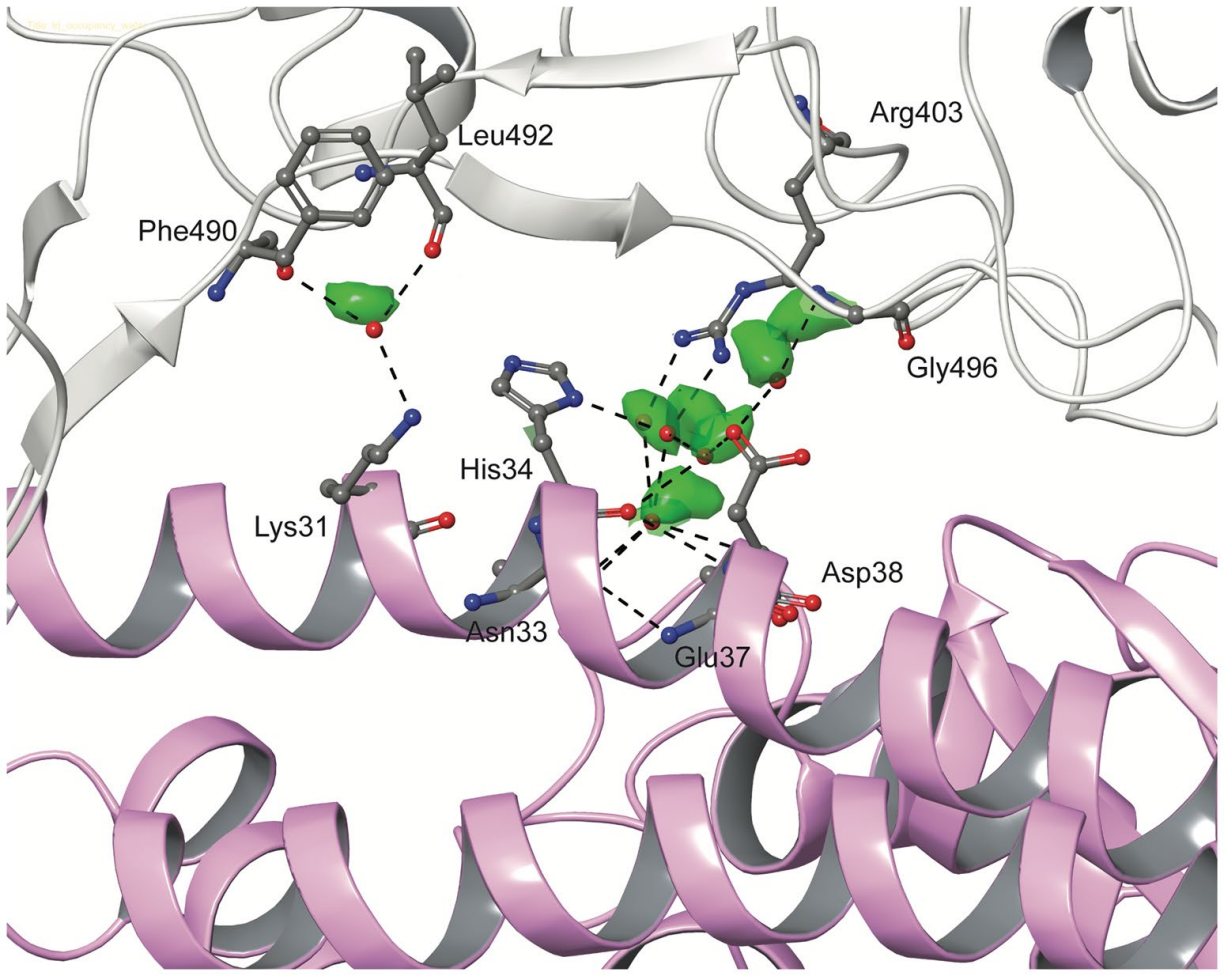
Conserved water sites in the interface between SARS-CoV-2 S RBD (grey) and ACE2 (pink) obtained from a 1 μs MD simulation of the complex. Regions with high residence time (green) were identified by constructing a 3D histogram of water occupancy sites. Specific water molecules from a frame of the simulation are shown as red spheres. For clarity only the oxygen atom of water molecules is shown. The interacting residues are shown in stick representation and hydrogen bonds are shown as black dashed lines.

Water molecules often play an important role in many intermolecular interfaces. In this instance, six conserved water sites were found in the interface between ACE2 and SARS-CoV-2 S RBD. Water-mediated indirect interactions were formed between ACE2:Lys31 and S:Phe490/Leu492, ACE2:Asp38 and S:Gly496, ACE2:Asn33/His34/Glu37/Asp38 and S:Arg403 (Fig. 6). These could also play a role in stabilizing the interface.

## Discussion

This study provides insight into the stability of the interactions that define the ACE2–SARS-CoV-2 and ACE2–SARS-CoV interfaces, using extended MD simulations of multiple structures of these complexes. Firstly, interactions that were shared by ACE2–SARS-CoV-2 and ACE2–SARS-CoV complexes were assessed. SARS-CoV-2 S protein RBD consistently interacted with ACE2 in three clusters. At one end, Tyr449, Gln493 and Gln498 formed sustained hydrogen bonds with Asp38, Glu35 and Lys353 of ACE2. At the other end Tyr473, Ala475 and Phe486 formed consistent hydrophobic interactions with Leu79, Met82 and Tyr83 of ACE2. In the middle, Lys417 formed a strong and stable salt bridge with Asp30, and Phe456 and Tyr489 formed sustained hydrophobic interactions with Thr27 of ACE2. Additionally, several intermittent interactions of SARS-CoV-2 could also permit it to bind more stably than SARS-CoV. These findings were also supported by the results from two different structures of the ACE2–SARS-CoV-2 S RBD complex (Fig. 5 and Supplementary Table S1 and S2). Loop regions (residues 484–505) of SARS-CoV-2 fluctuated less when bound to ACE2, when compared to SARS-CoV. This is stabilized by the formation of sustained interactions by Gln493 and Gln498 of SARS-CoV-2 with Glu35 and Lys353 of ACE2, respectively (Fig. 5A and Supplementary Figures S4 and S5).

Unlike SARS-CoV-2, in SARS-CoV, the region in the middle was devoid of any stable interactions with ACE2 (Fig. 4D). However, at the two ends, a different set of residues in SARS-CoV formed interactions with ACE2. Therefore, it is apparent that there are several similarities and differences in the structure and dynamics of the interactions of SARS-CoV-2 and SARS-CoV with ACE2. Hence, antibodies or antiviral treatment modalities that target the spike protein of SARS-CoV is not expected to produce a similar effect with SARS-CoV-2. Some of the recent studies that failed to inhibit the binding of SARS-CoV-2 RBD to ACE2, support these findings^13,17^.

Secondly, two charged virus binding hotspots on human ACE2 (Lys31 and Lys353), which are essential for the binding of SARS-CoV, have been studied extensively^4,8^. Charge neutralization of these hotspot lysines has been shown to be important for the binding of coronavirus to ACE2^7,8^. SARS-CoV-2 and SARS-CoV utilize unique strategies to achieve this. Interestingly, SARS-CoV-2 residues only formed sustained interactions with Lys353 of ACE2 and these were absent in SARS-CoV indicating an adaptation to a stronger interface (Fig. 5A,B). Several of these vital residues fall in loop regions of SARS-CoV-2 and SARS-CoV. Three loop regions—474–485, 488–490, and 494–505 of SARS-CoV-2 demonstrated limited fluctuation compared to the corresponding region in SARS-CoV (Fig. 3B,D). Lower fluctuations, observed in residues of SARS-CoV-2 that bind to the Lys353 hot spot of ACE2, could be another reason for the better affinity of SARS-CoV-2 to ACE2^7,11^.

Thirdly, the non-conserved residue Lys417 of SARS-CoV-2 formed a very stable salt bridge with Asp30 of ACE2. This interaction was absent in the corresponding residue (Val404) of SARS-CoV (Fig. 5 and Supplementary Table S1). Lys417 provides a positively charged patch on the RBD of SARS-CoV-2 which is absent in SARS-CoV. The RBD is also stabilized by this strong interaction; the RMSF of the region around Lys417 demonstrated lower fluctuations in SARS-CoV-2 RBD compared to the corresponding region in SARS-CoV (Fig. 3B,D). The strength of this sustained salt bridge between Lys417 and Asp30 could contribute to the substantially different binding affinity of SARS-CoV-2 for ACE2 when compared to SARS-CoV^11,20^. Notably, residues Leu455, Phe456, Tyr473, Phe486, Ser494 and Asn501, which were reported to form hydrogen bonds and electrostatic interactions leading to an enhanced binding affinity of SARS-CoV-2, were not observed in these simulations^3,7,20^.

Indeed, binding of the S protein to ACE2 is preferentially governed by hydrogen bonds and electrostatic interactions^11,22^. However, recent studies have reported the involvement of hydrophobic contacts in the interface that could influence the binding affinity for ACE2^23^. Here, the role of hydrophobic contacts between SARS-CoV-2/SARS-CoV and ACE2 was explored using MD simulations. Interestingly, SARS-CoV-2 RBD exhibited more sustained hydrophobic contacts with ACE2 than SARS-CoV S RBD. The formation of consistent hydrophobic interactions between Leu455, Phe456, Tyr473, Ala 475, Phe486 and Tyr489 of SARS-CoV-2 with the N-terminus residues of ACE2 such as Thr27, Phe28, Leu79, Met82 and Tyr83 contributes to the higher binding affinity of SARS-CoV-2. It is worth noting that these residues are mutated in SARS-CoV-2 RBD but for Tyr489. This is in agreement with studies that looked at hydrophobic contacts between SARS-CoV-2 and ACE2^23^. However, long MD simulations permit the evaluation and comparison of the stability of these interactions between SARS-CoV-2/SARS-CoV and ACE2.

The larger number of interactions in SARS-CoV-2 also translated to a notably higher ΔG_bind_, computed using MM-GBSA method. Hydrophobic contribution was also higher in the case of SARS-CoV-2.

In conclusion, while SARS-CoV-2 and SARS-CoV S RBD bind to the same region of ACE2 and share several similarities in how they interact with ACE2, there are a number of differences in the dynamics and energetics of the interactions. One salient difference is the presence of a stable salt bridge between Lys417 of SARS-CoV-2 S protein and Asp30 of ACE2 as well as three stable hydrogen bonds between Tyr449, Gln493 and Gln498 of SARS-CoV-2 and Asp38, Glu35 and Lys353 of ACE2, which were not observed in the ACE2–SARS-CoV interface. Stable viral binding with the host receptor is crucial for virus entry. Thus, special consideration should be given to these stable interactions while designing potential drugs and treatment modalities to target or disrupt this interface.

## Materials and methods

Coordinates of the three dimensional X-ray crystal structures of the SARS-CoV and SARS-CoV-2 RBD in complex with ACE2 were obtained from the Protein Data Bank (PDB). The PDB IDs of the structure used are 6M0J and 6LZG for SARS-CoV-2 S protein RBD bound to ACE2 and 2AJF for the SARS-CoV S protein RBD bound to ACE2. Schrödinger Maestro 2019-4 (Schrödinger, LLC, New York, NY) was used to visualize and prepare the protein structures for simulations. The structures were first pre-processed using the Protein Preparation Wizard (Schrödinger, LLC, New York, NY). The protein preparation stage included proper assignment of bond order, adjustment of ionization states, orientation of disorientated groups, creation of disulphide bonds, removal of unwanted water molecules, metal and co-factors, capping of the termini, assignment of partial charges, and addition of missing atoms and side chains. In the case of the SARS-CoV structure, a loop (residues 376–381) missing in the PDB structure was modelled using Schrödinger Prime^24^. Hydrogen atoms were incorporated, and standard protonation state at pH 7 was used. Structures of spike protein RBD bound to ACE2 were placed in orthorhombic boxes of size 125 Å × 125 Å × 125 Å and solvated with single point charge (SPC) water molecules using the Desmond System Builder (Schrödinger, LLC, New York, NY). A box size of 85 Å × 85 Å × 85 Å was used for simulations of S RBD structures isolated from the PDB structures 6M0J (chain E) and 2AJF (chain E). Simulation systems were neutralized with counterions and a salt concentration of 0.15 M NaCl was maintained. MD simulations were performed using Desmond^25^. The OPLS forcefield was used for all calculations. All systems were subjected to Desmond’s default eight stage relaxation protocol before the start of the production run. Initially, 500 ns simulations were performed in triplicate with a different set of initial velocities for simulations involving 6M0J, 6LZG, and 2AJF chains A/E. Two additional 500 ns simulations of 2AJF chains B/F were also performed. Subsequently, one simulation each of 6M0J, 2AJF chains A/E and 2AJF chains B/F were extended to 1 μs to ensure that the interactions were retained for a longer duration. One 500 ns simulation each of just the S protein isolated from 6M0J and 2AJF were also run. For the simulations, the isotropic Martyna–Tobias–Klein barostat and the Nose–Hoover thermostat were used to maintain the pressure at 1 atm and temperature at 300 K, respectively^26,27^. Short-range cutoff was set as 9.0 Å and long-range coulombic interactions were evaluated using the smooth particle mesh Ewald method (PME)^28^. A time-reversible reference system propagator algorithm (RESPA) integrator was employed with an inner time step of 2.0 fs and an outer time step 6.0 fs^29^. MM-GBSA method was used to evaluate the free energy of binding of S protein RBD to ACE2 using frames extracted from MD simulation trajectories. Frames were extracted every 10 ns from each of the simulations and MM-GBSA based binding free energy was computed using Schrödinger Prime employing the VSGB 2.0 solvation model^30^. Simulation data was analysed using packaged and in-house scripts. Graphs were plotted using R version 3.6.3 (https://www.r-project.org) and images of structures were generated using Visual Molecular Dynamics version 1.9.3^31^.

## Supplementary information

Supplementary Information.

## Data Availability

The datasets generated during and/or analysed during the current study are available from the corresponding author on reasonable request.
