# Supplementary material for: Dynamics of the ACE2–SARS-CoV-2/SARS-CoV spike protein interface reveal unique mechanisms
Source: Sci Rep. 2020 Aug 26;10:14214. doi: 10.1038/s41598-020-71188-3 (PMC7449962; doi:10.1038/s41598-020-71188-3)
Supplement: Supplementary file 1 — Supplementary Information. [file 41598_2020_71188_MOESM1_ESM.pdf]

Dynamics of the ACE2 - SARS-CoV-2/SARS-CoV spike protein interface reveal unique mechanisms

Amanat Ali and Ranjit Vijayan*

Department of Biology, College of Science, United Arab Emirates University, PO Box 15551, Al Ain, United Arab Emirates.

* Correspondence: Ranjit Vijayan

Tel: +971 3 7136302

Email: ranjit.v@uaeu.ac.ae

Supplementary Information

Table S1. Percentage of simulation time polar interactions were formed between ACE2 residues and residues in equivalent positions of SARS-CoV-2 and SARS-CoV S RBD. For e.g., Lys417 of SARS-CoV-2 S protein RBD is in the same position as Val404 of SARS-CoV S RBD. For equivalent positions, see Figure 1.

**ACE2 residue**	**SARS-CoV-2 spike protein residue**	**Contact time (%) (PDB ID: 6M0J)**			**Contact time (%) (PDB ID: 6LZG)**			**SARS-CoV spike protein residue**	**Contact time (%) (PDB ID: 2AJF, chains A/E)**			**Contact time (%) (PDB ID: 2AJF, chains B/F)**	
		**Run1**	**Run2**	**Run3**	**Run1**	**Run 2**	**Run3**		**Run1**	**Run2**	**Run3**	**Run1**	**Run2**
**Asp30**	**Lys417**	93.2	96.3	98.6	98.0	92.8	86.4	**Val404**	Nil	Nil	Nil	Nil	Nil
**Glu35**	**Gln493**	82.3	81.3	91.2	90.3	86.0	84.6	**Asn479**	11.7	11.5	16.4	3.9	Nil
**Asp38**	**Tyr449**	54.8	88.6	80.8	10.0	13.2	45.9	**Tyr436**	10.0	6.5	2.7	3.3	2.6
**Lys353**	**Gln498**	73.5	95.3	95.5	90.7	22.3	90.7	**Tyr484**	1.0	0.6	0.5	0.4	0.6
**Gln24**	**Tyr489**	2.8	48.7	61.1	0.04	24.5	22.2	**Tyr475**	Nil	Nil	Nil	Nil	Nil
**Asp38**	**Gln498**	33.6	95.4	81.5	84.6	57.4	95.6	**Tyr484**	2.0	4.1	0.4	0.7	0.4
**Lys353**	**Gly496**	79.9	37.6	55.6	83.6	11.6	28.8	**Gly482**	12.0	4.4	1.8	2.2	1.8
**Thr27**	**Tyr489**	0.77	91.8	91.8	0.06	0.02	13.2	**Tyr475**	Nil	0.2	Nil	0.13	Nil
**His34**	**Tyr453**	39.0	23.9	83.1	36.1	29.4	12.6	**Tyr440**	0.01	Nil	20.3	Nil	20
**Glu37**	**Tyr505**	88.5	3.8	1.1	71.0	36.1	6.6	**Tyr491**	17.8	9.85	61.1	71.6	69.31
**Gln42**	**Gly446**	15.8	55.5	40.3	0.04	10.1	37.7	**Thr433**	0.15	2.5	Nil	Nil	Nil
**Lys353**	**Gly502**	99.9	16.0	26.7	99.8	6.7	2.4	**Gly488**	99.5	96.7	99.3	99.5	97.0
**Asp355**	**Thr500**	52.3	1.6	18.5	56.9	4.7	1.5	**Thr486**	99.1	97.9	98.5	99.3	96.1
**His34**	**Lys417**	55.6	0.23	0.2	34.9	54.6	25.2	**Val404**	Nil	Nil	Nil	Nil	Nil
**Lys31**	**Leu455**	Nil	Nil	Nil	Nil	Nil	Nil	**Tyr442**	86.2	1.2	3.5	4.6	2
**Tyr83**	**Phe486**	8.6	26.8	29.2	17.9	25.6	29.1	**Leu472**	Nil	Nil	Nil	Nil	Nil
**Lys31**	**Tyr489**	0.3	0.02	Nil	2.1	0.2	0.2	**Tyr475**	70.6	0.76	1.9	6.9	1.1
**Gln24**	**Ala475**	30.6	18.4	35.7	27.5	10.7	10.6	**Pro462**	Nil	0.2	Nil	0.12	Nil
**Tyr41**	**Thr500**	35.2	0.75	0.22	Nil	1.5	92.3	**Thr486**	0.41	Nil	Nil	Nil	Nil
**Lys31**	**Glu484**	30.5	21.5	39.52	15.2	6.3	6.7	**Pro470**	Nil	0.05	Nil	Nil	Nil
**Lys31**	**Gln493**	41.8	42.8	21.9	54.5	57.6	62.5	**Asn479**	1.7	Nil	0.5	Nil	Nil

Table S2. Percentage of simulation time hydrophobic interactions were formed between ACE2 residues and residues in equivalent positions of SARS-CoV-2 and SARS-CoV S RBD. For e.g., Phe486 of SARS-CoV-2 S protein RBD is in the same position as Leu472 of SARS-CoV S RBD. For equivalent positions, see Figure 1.

ACE2 residue	SARS-CoV-2 spike protein residue	Contact time (%) (PDB ID: 6M0J)			Contact time (%) (PDB ID: 6LZG)			SARS-CoV spike protein residue	Contact time (%) (PDB ID: 2AJF, chains A/E)			Contact time (%) (PDB ID: 2AJF, chains B/F)	
		**Run1**	**Run2**	**Run3**	**Run1**	**Run2**	**Run3**		**Run1**	**Run2**	**Run3**	**Run1**	**Run2**
**Leu79**	**Phe486**	99.2	98.4	99.7	97.7	99.0	99.4	**Leu472**	6.9	3.8	0.89	1.2	1.9
**Met82**	**Phe486**	99.7	99.9	99.9	99.2	98.7	99.9	**Leu472**	Nil	Nil	Nil	Nil	Nil
**Tyr83**	**Phe486**	98.8	99.8	99.8	99.4	99.5	99.7	**Leu472**	1.2	0.7	0.3	0.03	2.2
**Phe28**	**Tyr489**	99.9	99.9	99.9	99.9	99.9	99.6	**Tyr475**	25.3	18	2.3	7.2	5.4
**Tyr83**	**Tyr489**	78.7	95.8	98.9	61.4	78.1	79.4	**Tyr475**	0.54	7.2	3.4	0.7	2.2
**Thr27**	**Phe456**	98.6	99.9	99.9	99.9	93.7	99.9	**Leu443**	Nil	Nil	Nil	Nil	Nil
**Thr27**	**Tyr489**	90.9	97.7	97.7	96.4	98.0	99.5	**Tyr475**	Nil	Nil	Nil	Nil	Nil
**Thr27**	**Ala475**	77.4	90.4	94.9	77.9	70.3	87.6	**Pro462**	Nil	Nil	Nil	Nil	Nil
**Thr27**	**Tyr473**	66.4	98.5	96.5	79.3	76.7	99.4	**Phe460**	Nil	Nil	Nil	Nil	Nil
**Tyr41**	**Gln498**	Nil	Nil	Nil	Nil	Nil	Nil	**Tyr484**	97.1	98.3	96.6	98.8	80.8
**Leu45**	**Gln498**	Nil	Nil	Nil	Nil	Nil	Nil	**Tyr484**	83.4	36.3	77.2	65.8	48.9
**Phe28**	**Ala475**	Nil	Nil	Nil	Nil	Nil	0.003	**Pro462**	98.9	0.05	Nil	11	0.02
**Leu79**	**Ala475**	Nil	Nil	Nil	Nil	Nil	Nil	**Pro462**	88.4	Nil	Nil	0.05	0.05
**Phe32**	**Tyr489**	Nil	Nil	Nil	Nil	Nil	Nil	**Tyr475**	56	Nil	Nil	Nil	Nil
**Phe72**	**Tyr489**	Nil	Nil	Nil	Nil	Nil	Nil	**Tyr475**	83.9	Nil	Nil	0.03	Nil

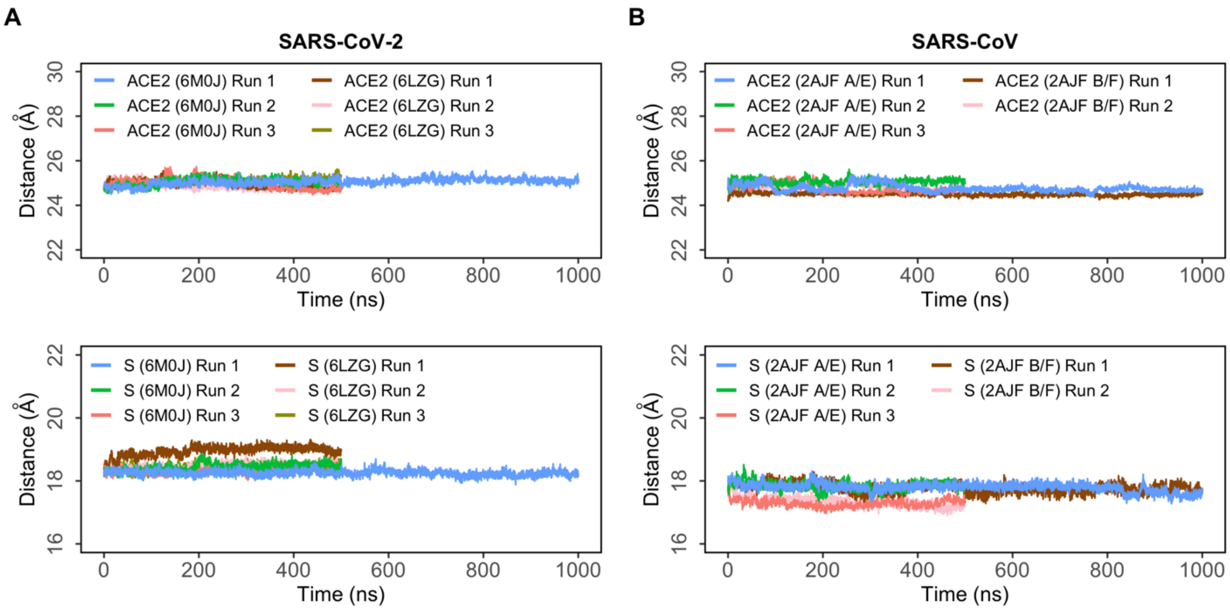

Figure S1. Radius of gyration (Rg) of ACE2 and spike (S) protein of SARS-CoV-2 and SAR-CoV. Six simulations of the ACE2-SARS-CoV-2 complex were performed using two structures (6M0J and 6LZG). Five simulations of ACE2-SARS-CoV complex were performed; three using chains A and E of structure 2AJF and two using chains B and F. All simulations were run for at least 500 ns while the first simulation of the ACE2-SARS-CoV-2 complex (6M0J), and the first simulation of chains A/E and B/F of ACE2-SARS-CoV complexes were extended up to 1 µs. A) Top: Rg of ACE2 in ACE2-SARS-CoV-2 complex, Bottom: Rg of S in ACE2-SARS-CoV-2 complex; B) Top: Rg of ACE2 in ACE2SARS-CoV- complex, Bottom: Rg of S in ACE2-SARS-CoV complex.

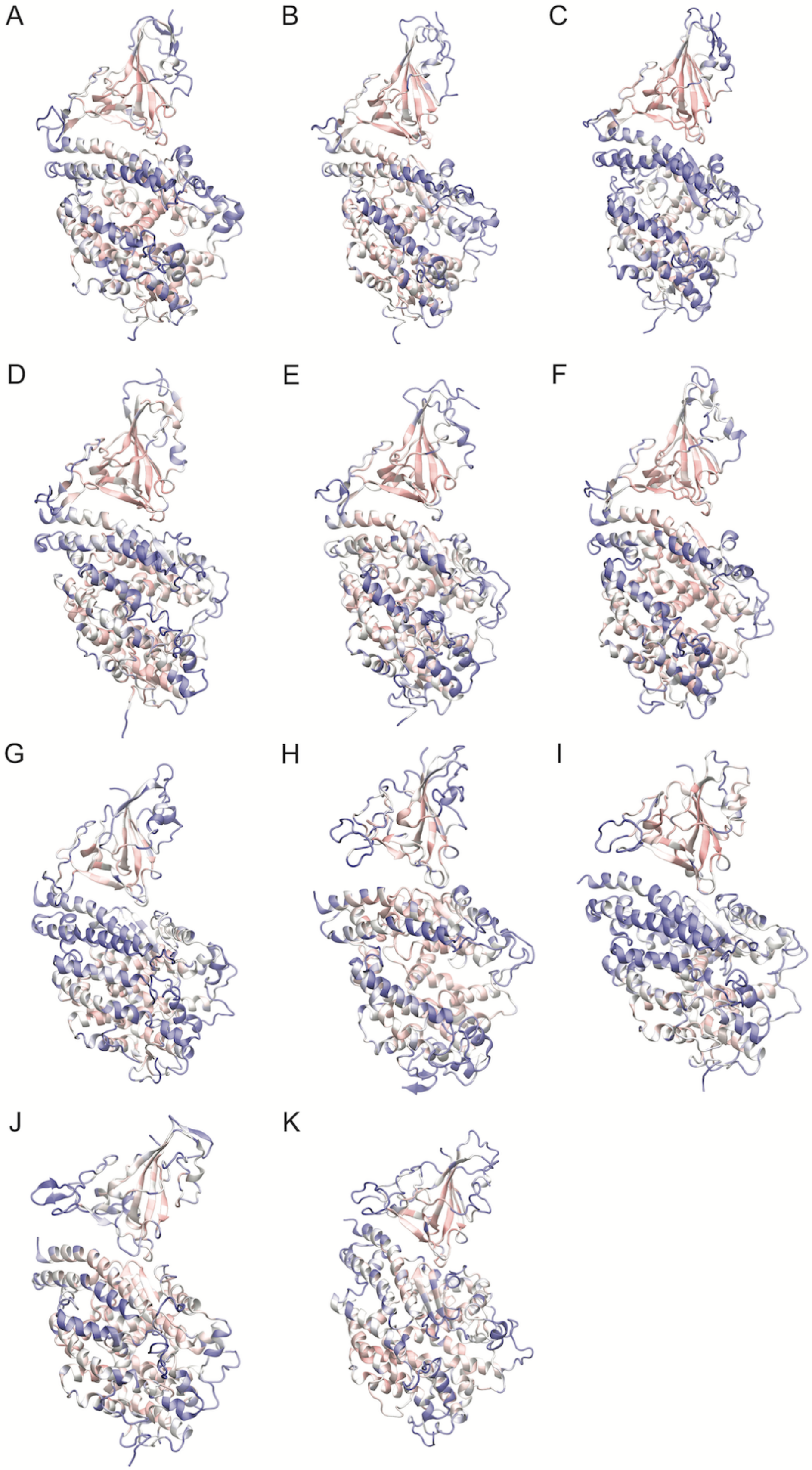

Figure S2. RMSF projected as beta factors to representative structures of ACE2-SARS-CoV-2 and ACE2-SARS-CoV complexes. Pink shows regions of low flexibility and blue shows regions of high flexibility.

A-C) ACE2-SARS-CoV-2 complex in three runs of 6M0J;

D-F) ACE2-SARS-CoV-2 complex in three runs of 6LZG;

G-I) ACE2-SARS-CoV complex in three runs of 2AJF chains A/E;

J-K) ACE2-SARS-CoV complex in two runs of 2AJF chains B/F.

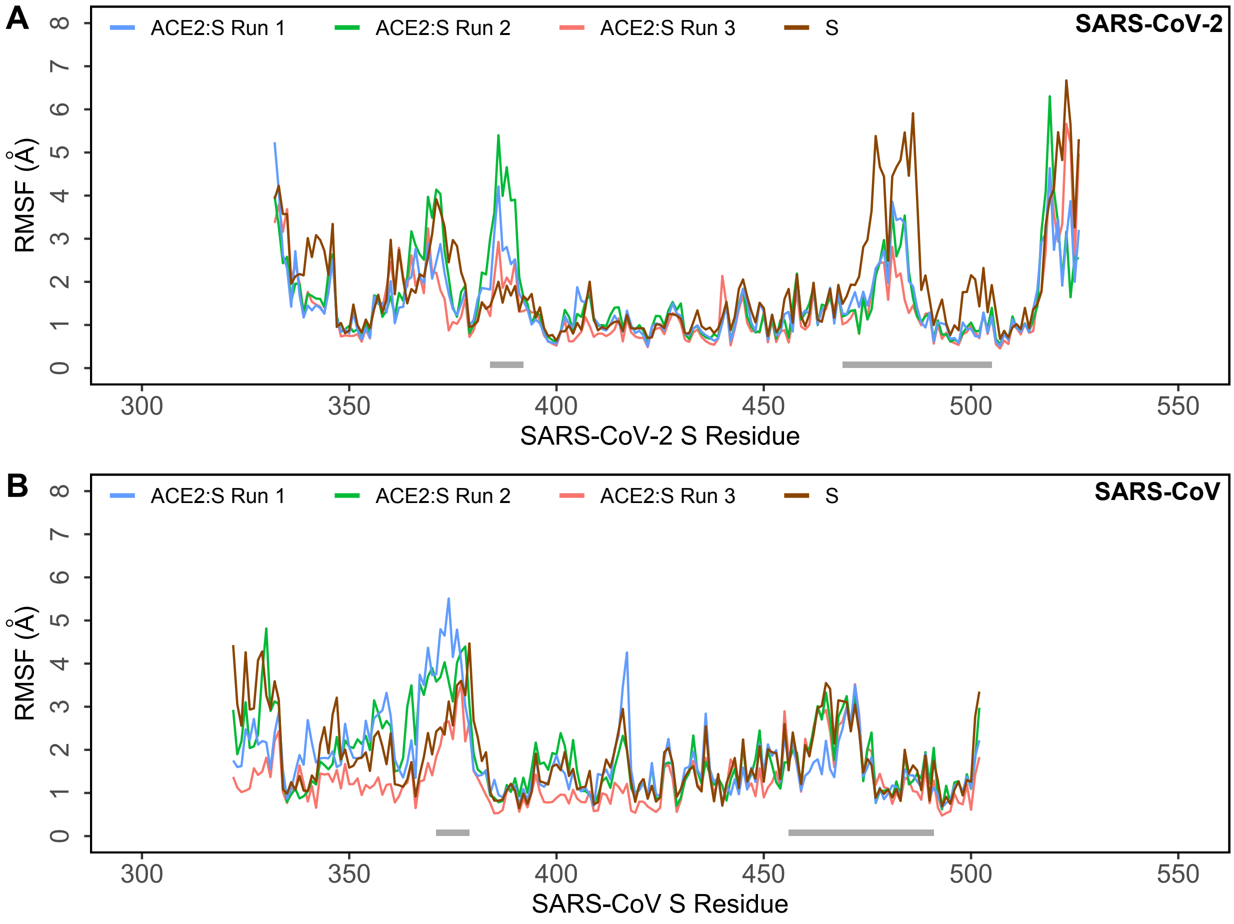

Figure S3. Root mean square fluctuation (RMSF) of spike (S) protein RBD Cα atoms obtained from three independent runs (1 × 1 µs and 2 × 500 ns) of SARS-CoV-2 and SARS-CoV S protein bound to ACE2 compared to 500 ns unbound S only simulations of SARS-CoV-2 and SARS-CoV. A) RMSF of Cα atoms of SARS-CoV-2 S protein RBD in complex with ACE2 and without ACE2; B) RMSF of Cα atoms of SARS-CoV S protein RBD in complex with ACE2 and without ACE2. Notable difference in RMSF of loop regions of SARS-CoV-2 and SARS-CoV are marked with a grey line.

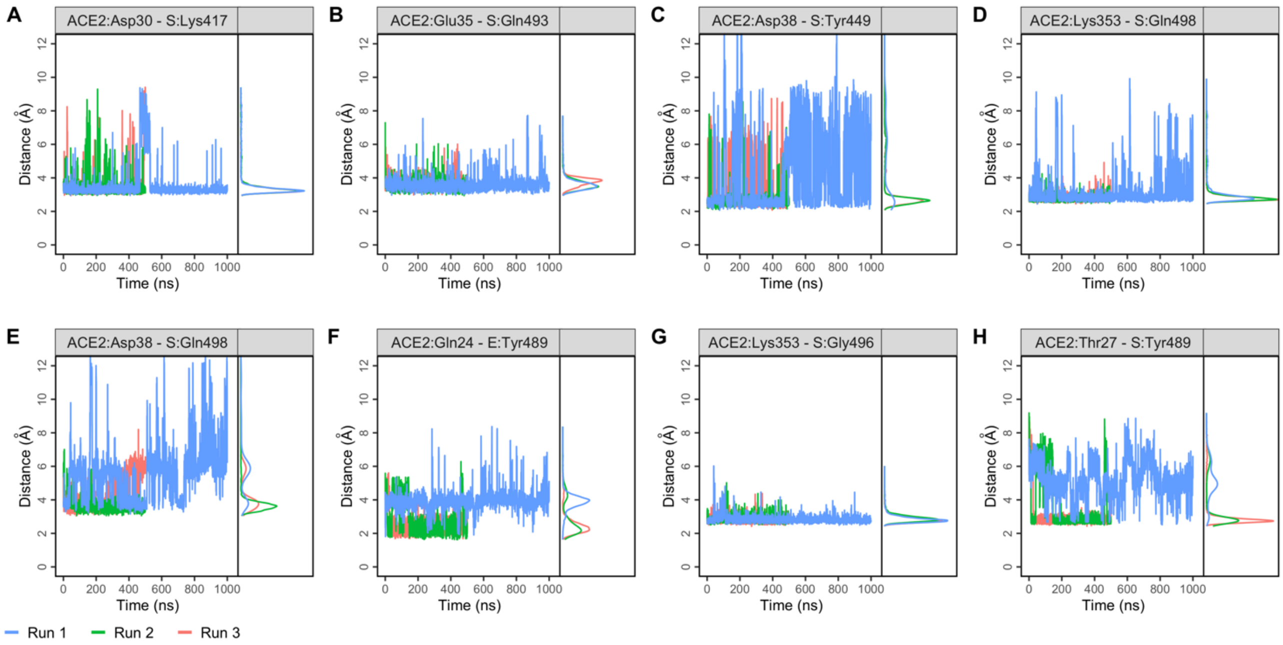

Figure S4. Contact distance plots of interfacial polar interactions between ACE2 and SARS-CoV-2 spike (S) protein RBD from three simulations of 6M0J. The contact distance was evaluated between specific atoms as indicated below to account for the possibility of switches between different hydrogen atoms (in Lys) or oxygen atoms (in Asp/Glu) that is involved in a hydrogen bond. Data from the three runs are shown in red, green, and blue. The contact distance density plot of consistently interacting residues showed sharper peaks in the density plot adjacent to each distance plot. A) Distance between ACE2:Asp30_CG and S:Lys417_NZ; B) Distance between ACE2:Glu35_CD and S:Gln493_NE2; C) Distance between ACE2:Asp38_CG and S:Tyr449_HH; D) Distance between ACE2:Lys353_NZ and S:Gln498_OE1; E) Distance between ACE2:Asp38_CG and S:Gln498_NE2; F) Distance between ACE2:Gln24_O (backbone) and S:Tyr489_HH; G) Distance between ACE2:Lys353_NZ and S:Gly496_O (backbone); H) Distance between ACE2:Thr27_OG1 and S:Tyr489_OH.

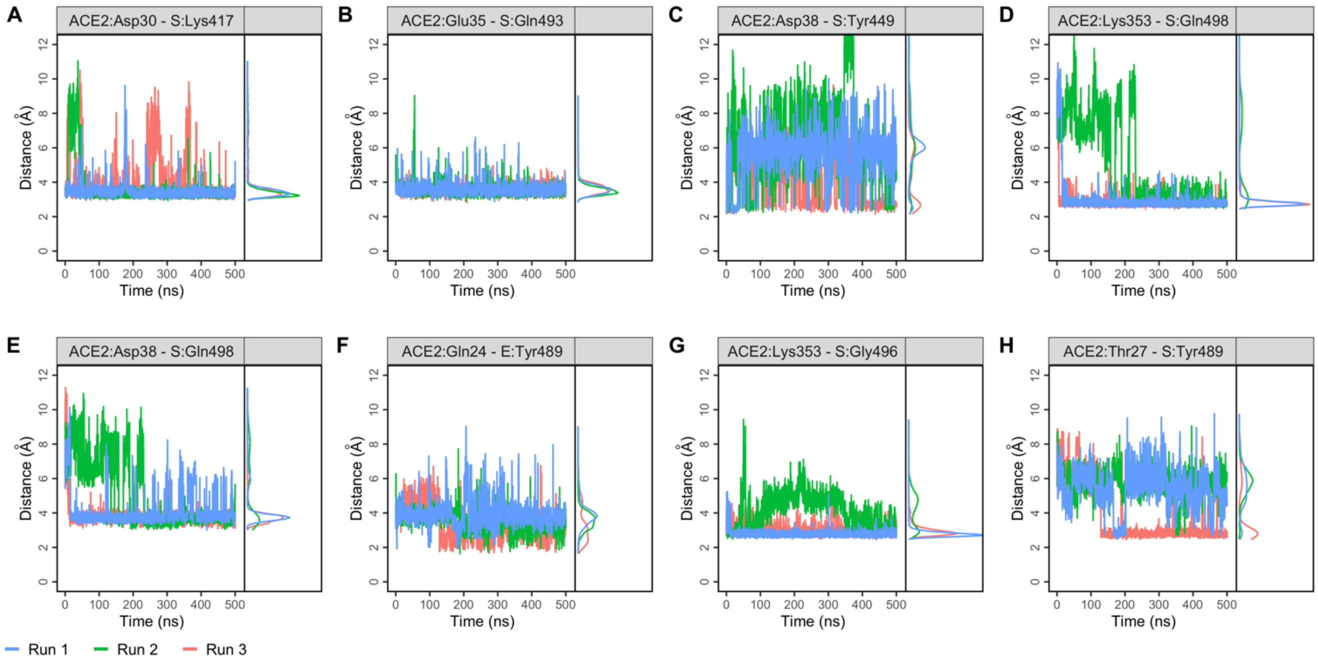

Figure S5. Contact distance plots of interfacial polar interactions between ACE2 and SARS-CoV-2 spike (S) protein RBD from three simulations of 6LZG. The contact distance was evaluated between specific atoms as indicated below to account for the possibility of switches between different hydrogen atoms (in Lys) or oxygen atoms (in Asp/Glu) that is involved in a hydrogen bond. Data from the three runs are shown in red, green, and blue. The contact distance density plot of consistently interacting residues showed sharper peaks in the density plot adjacent to each distance plot. A) Distance between ACE2:Asp30_CG and S:Lys417_NZ; B) Distance between ACE2:Glu35_CD and S:Gln493_NE2; C) Distance between ACE2:Asp38_CG and S:Tyr449_HH; D) Distance between ACE2:Lys353_NZ and S:Gln498_OE1; E) Distance between ACE2:Asp38_CG and S:Gln498_NE2; F) Distance between ACE2:Gln24_O (backbone) and S:Tyr489_HH; G) Distance between ACE2:Lys353_NZ and S:Gly496_O (backbone); H) Distance between ACE2:Thr27_OG1 and S:Tyr489_OH.

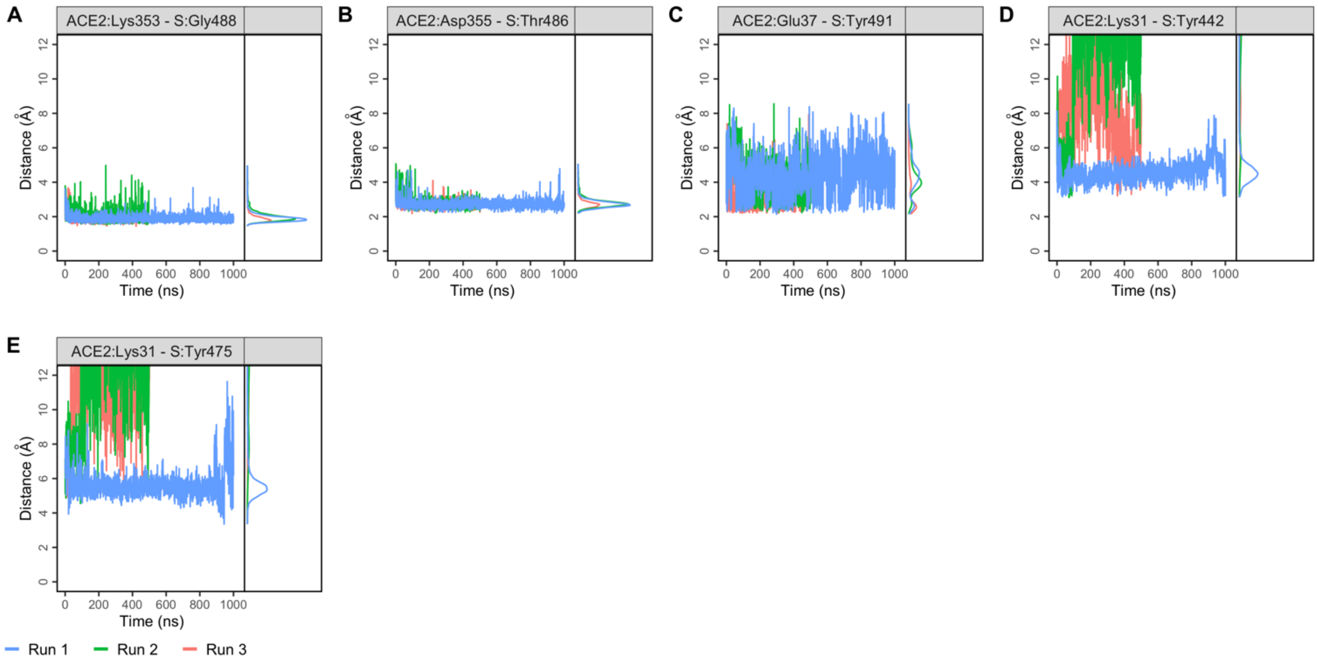

Figure S6. Contact distance plots of interfacial polar interactions between ACE2 and SARS-CoV spike (S) protein RBD from three simulations of chains A/E of the structure 2AJF. Data from the three runs are shown in red, green, and blue. The contact distance density plot of consistently interacting residues showed sharper peaks in the density plot adjacent to each distance plot. A) Distance between ACE2:Lys353_O (backbone) and S:Gly488_H (backbone); B) Distance between ACE2:Asp355_CG and S:Thr486_HG1; C) Distance between ACE2:Glu37_CD and S:Tyr491_HH; D) Distance between ACE2:Lys31_NZ and S:Tyr442_CZ (cation-π); E) Distance between ACE2:Lys31_NZ and S:Tyr475_CZ (cation-π).

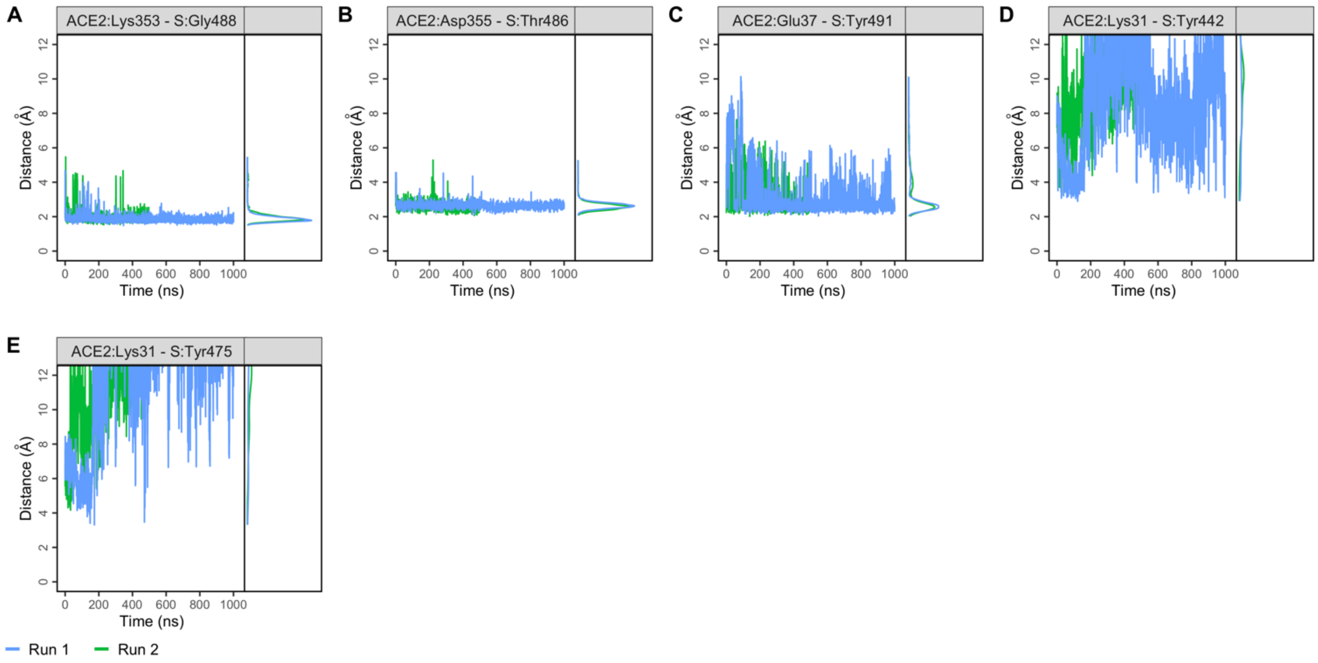

Figure S7. Contact distance plots of interfacial polar interactions between ACE2 and SARS-CoV spike (S) protein RBD from three simulations of chains B/F of the structure 2AJF. Data from the three runs are shown in red, green, and blue. The contact distance density plot of consistently interacting residues showed sharper peaks in the density plot adjacent to each distance plot. A) Distance between ACE2:Lys353_O (backbone) and S:Gly488_H (backbone); B) Distance between ACE2:Asp355_CG and S:Thr486_HG1; C) Distance between ACE2:Glu37_CD and S:Tyr491_HH; D) Distance between ACE2:Lys31_NZ and S:Tyr442_CZ (cation-π); E) Distance between ACE2:Lys31_NZ and S:Tyr475_CZ (cation-π).

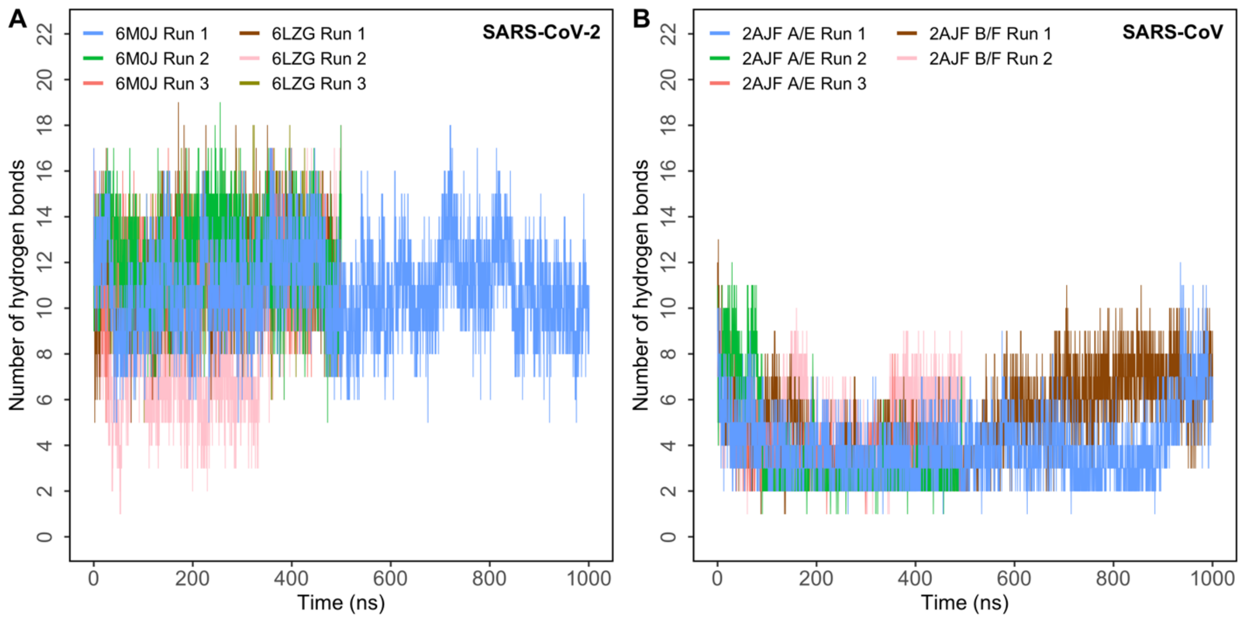

Figure S8. Number of intermolecular hydrogen bonds formed between ACE2 and SARS-CoV-2/SARS-CoV spike (S) protein RBD. A) Number of hydrogen bonds formed between ACE2 and SARS-CoV-2 S from three simulations (1 × 1 μs and 2 × 500 ns) of 6M0J and three simulations of (3 × 500 ns) of 6LZJ structures; B) Number of hydrogen bonds formed between ACE2 and SARS-CoV S from three simulations (1 × 1 μs and 2 × 500 ns) of chains A/E, and two simulations (1 × 1 μs and 1 × 500 ns) of chains B/F of 2AJF.
